# Work situation and self-perceived economic situation as predictors of change in burnout – a prospective general population-based cohort study

**DOI:** 10.1186/s12889-015-1681-x

**Published:** 2015-04-03

**Authors:** Sofia Norlund, Christina Reuterwall, Jonas Höög, Urban Janlert, Lisbeth Slunga Järvholm

**Affiliations:** Department of Public Health and Clinical Medicine, Occupational and Environmental Medicine, Umeå University, Umeå, Sweden; Department of Sociology, Umeå University, Umeå, Sweden; Department of Public Health and Clinical Medicine, Epidemiology, Umeå University, Umeå, Sweden

**Keywords:** Psychosocial, Occupational, Mental disorder, Exhaustion, General population, Sweden

## Abstract

**Background:**

Sick leave rates due to mental and behavioural disorders have increased in Sweden during the last decades. The aim of this prospective study was to investigate changes in the level of burnout in a working subset of the general population and to identify how such changes relate to changes in work situation and self-perceived economic situation.

**Methods:**

A cohort of 1000 persons from a subset of the 2004 northern Sweden MONICA (Multinational Monitoring of Trends and Determinants in Cardiovascular Disease) general population survey was followed over a five-year period (2004–2009). In total, 623 persons (323 women and 300 men) were included in the analysis. Burnout levels were measured at baseline and follow-up using the Shirom Melamed Burnout Questionnaire. Risk factors were assessed at both measuring points.

**Results:**

In the whole study cohort, a small (−0.15) but statistically significant reduction in burnout level was found. No differences in change of burnout were found between men and women. Constant strain at work, an increased risk of unemployment, and a perceived worsening of economic situation during the study time period were related to an increased burnout level. An accumulation of these risk factors was associated with increased burnout level.

**Conclusions:**

Risk factors in work situation and self-perceived economy are related to changes in burnout level, and special attention should be directed towards persons exposed to multiple risk factors.

## Background

Mental health disorders are a significant social problem and are among the leading causes for absenteeism from work and for disability pension [1,2]. Employment is usually associated with good mental health, but it can also be harmful if employment conditions are poor [3]. The labour market has changed in recent decades with increased competition, and many people today experience increasing work demands and a high degree of job insecurity [4].

Burnout was originally described as a phenomenon characterized by emotional exhaustion, depersonalisation, and reduced personal accomplishment that affected people working in client-related occupations. The concept of burnout has since been broadened and is now most often considered to be a state that develops from chronic stress, and is manifested through physical and mental fatigue, emotional exhaustion, and cognitive problems [5]. Burnout is often considered a predictor for disability pension, all-cause sick leave, and all-cause mortality [6-8]. In Sweden, the new diagnosis Exhaustion Disorder has been developed, which is closely related to the burnout concept [9]. Exhaustion Disorder is a common mental health problem in Sweden and is frequently reported as a cause of sick leave from work [10].

The psychosocial work climate is important for people’s well being and has been found to predict sick leave and disability pension [11,12]. Studies have shown associations between psychosocial work environment and burnout [13-15], and job demands and control are commonly studied psychosocial work factors associated with burnout [16-18]. Lindeberg et al. [19] found in a one-year longitudinal study that high demands and low control predicted exhaustion in a working population and that the combination of high demands and low control at work, referred to as job strain, could work synergistically to increase the risk of exhaustion. Another Swedish study [20] on a working population found that over a three-year period high demands predicted symptoms of emotional exhaustion in the whole population and that low control was a significant predictor of exhaustion in women.

Job insecurity has been associated with the risk of poor physical and mental health [21-23]. In the literature on burnout, the impact of being exposed to job insecurity has mostly been discussed in the form of downsizing [20,24,25]. A person’s economic situation can be important for their state of mental health [26,27], but to our knowledge self-perceived economic situation and burnout have not previously been studied longitudinally.

In a cross-sectional study in northern Sweden in 2004, we assessed the level of burnout in the working segment of the general population and studied associations between burnout and working conditions [28]. Demands and control at work, job insecurity, and self-perceived economic situation were all associated with burnout. Because of the cross-sectional design of that study, however, causal effects could not be investigated.

To our knowledge, there is only a small number of prospective studies regarding work-related exposure and burnout. Some studies have examined changes in exposure [19,29] while others have examined the predictive capacity of baseline exposure levels [13,20]. Some have adjusted for baseline burnout [13], although most studies have not [19,20,29]. Few studies have comprised general working populations [19,20], and we found only one prospective study that simultaneously examined changes in work-related exposure and changes in burnout/emotional exhaustion [30].

In this prospective cohort study, our aims were to study individual changes in level of burnout over a five-year period (between 2004 and 2009) in an occupationally active subset of the general population and to study relations between changes in job strain, job insecurity, and self-perceived economic situation and changes in burnout level.

## Methods

### Study population

This is a longitudinal study based on a subset of the 2004 northern Sweden MONICA (Multinational Monitoring of Trends and Determinants in Cardiovascular Disease) survey screening of the general population [31]. A total of 1000 persons (497 women and 503 men) in the 2004 survey screening met specific criteria and were studied in a cross-sectional burnout study [28]. The inclusion criteria were being under 65 years of age, occupationally active, not on sick leave, and having no more than one missing item on the Shirom Melamed Burnout Questionnaire (SMBQ). Written informed consent for participation in the study was obtained from all participants. Ethical approval was granted by the Regional Ethical Review Board in Umeå (§464/03, Dnr 03–375).

In the spring of 2009, a follow-up survey was sent to the participants in the cross-sectional burnout study. The same questions as in the 2004 survey were incorporated in the 2009 survey. A postal questionnaire and an information letter were sent to each participant. We sent out three reminders at one-month intervals. Persons who had retired or received a disability pension were excluded from all analyses. Unemployed persons were excluded from certain analyses (see the Results section).

This follow-up study was approved by the Regional Ethical Review Board in Umeå (Dnr 08-051 M).

### Outcome variable – change in burnout level

Burnout was operationalized in this study according to the criteria for burnout developed by Shirom and Melamed [5], and the SMBQ [32] was used to estimate the level of burnout. The instrument measures a general burnout state in life and not occupational burnout in particular. The questionnaire consists of 22 statements, for example, “I feel physically fatigued” and “I have trouble concentrating”. Each item is scored on a 7-point frequency scale ranging from 1 (almost never) to 7 (almost always). For each respondent, an SMBQ score is calculated as the mean of all 22 items. Giving answers to at least 21 of the 22 statements was an inclusion criterion. If one item was missing (n = 39), the mean value of all other respondents’ scores on that particular question was imputed. For each individual, the SMBQ score change was calculated as the SMBQ score in 2009 minus the SMBQ score in 2004. A reduction in SMBQ score between the two measuring points (the favourable outcome) gives a negative value for SMBQ score change. Change in SMBQ score is also referred to as change in burnout level.

### Change in risk factors

The job demand-control model [33] was incorporated into the study to assess the psychosocial work environment. The risk factor *job strain* is made up of two dimensions, demands and control, that contain five and six items respectively [28]. Individuals who experienced high demands simultaneously with low control were considered to be exposed to job strain. The dimensions were dichotomized based on percentiles. For demands, all responders above the 75^th^ percentile were considered to have “high demands”, and for control, all responders below the 25^th^ percentile were considered to have “low control”. The cut-off values from the 2004 data were applied to the 2009 data.

Job insecurity was measured by two factors that stem from one question each. *Risk of unemployment* was assessed by the question “Are you at risk of becoming unemployed in the near future?” with the answer alternatives “yes” and “no”. The variable *new job possibilities* was assessed with the question “If you lost your job, what would your chances be of getting a new job within a month?” and was dichotomized into a lower (“no chance” and “small”) and a higher (“rather good” and “very good”) chance.

To assess the respondents’ *self-perceived economic situation*, the question “How satisfied are you with your economic situation?” was answered using a 7-point scale ranging from 1 (very dissatisfied) to 7 (very satisfied). The variable was dichotomized on the lower quartile (the same value as in 2004) creating two groups labelled dissatisfied (with a score of 1–4) or satisfied (with a score of 5–7).

Because each of the risk factors was measured in both 2004 and 2009, the changes in each respondent’s exposure level could be observed. After dichotomization, each factor had two possible exposure levels at both measuring points. Thus, there were four possible outcomes: experiencing a constant favourable situation, experiencing a constant unfavourable situation, and changing from a better to a worse situation or vice versa.

### Potential confounders

The following four potential confounders were considered: age, sex, socioeconomic index (SEI), and social integration. SEI was categorized according to occupation as blue-collar, white-collar, and self-employed. Social integration was measured using four questions from the Interview Schedule of Social Interaction Scale (ISSI). The questions are part of the Availability of Social Integration (AVSI) section of the scale and measure the number of persons in an individual’s social support network. Subjects whose answers indicate at least three friends for all items were labelled as having good social support [34].

The selected potential confounders were chosen on the basis of the associations found in the previous cross-sectional study [28]. Measures from 2009 were used in the present study.

### Statistical analysis

The data analysis was carried out using PASW Statistics 18 (formerly known as SPSS statistics) with the level of significance set at <0.05. Characteristics of the study population at baseline and at the 5-year follow-up were analysed by using paired-samples *t*-test, the McNemar chi-square test, and the marginal homogeneity test. Results of McNemar chi-square tests are presented without correction for continuity. When comparing SMBQ score change in different age groups, the p-values from one-way ANOVAs were used. Linear regression analysis was used to test the SMBQ score change in relation to each single risk factor. For each factor, the category “constant favourable situation” served as the reference category. The four potential confounders were included in these regression analyses as adjustments. Linear regression analysis was also used when comparing the number of risk factors that the individual was exposed to.

## Results

**Figure 1:**
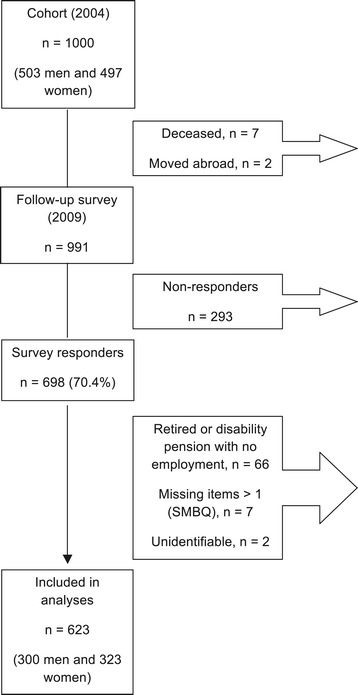
**A flowchart for description of the study cohort originating from the 2004 northern Sweden MONICA (Multinational Monitoring of Trends and Determinants in Cardiovascular Disease) general population survey.**

A total of 698 persons responded to the 2009 survey giving a response rate of 70.4% (Figure 1). A total of 75 persons were excluded from the analyses due to retirement, disability pension without continued employment, more than one missing value on the SMBQ, or an unidentifiable ID number, and this meant that 623 persons (300 men and 323 women) were included in the analyses. At follow-up, 22 persons were on some sort of sick leave (18 of the 22 persons were employed), 14 persons had become unemployed, and 3 persons had become students. Due to the nature of the questions, unemployed persons were not included in analyses regarding job strain and risk of unemployment. They were also excluded from the analysis regarding the number of risk factors and the change in burnout level.

Non-responders and participants were compared with respect to their SMBQ scores in 2004. There was no significant statistical difference between the overall mean scores of the two groups (2.89 and 2.85 for non-responders and participants, respectively). Some differences were found between female non-responders and women who participated in the study. Female non-responders were to a larger extent blue-collar (51%) compared with participants (34%), more female non-responders perceived a lower degree of control at work (36% compared with 25%), and more female non-responders expressed a higher risk of becoming unemployed (19%) than female participants (11%).

Characteristics of the final study population at baseline and at the 5-year follow-up are presented in Table 1. The SMBQ mean score was significantly reduced from 2.86 (SD 0.91) to 2.71 (SD 1.05) during this time period, and fewer persons reported living with children, more persons estimated low new job possibilities, and more were dissatisfied with their economic situation at the follow-up measurement. Approximately 12-13% of the study population had an SMBQ level exceeding 4.0, which has previously been used as a cut-off indicating a clinical burnout state [10].

**Table 1:** **Characteristics of the study sample consisting of 300 men and 323 women from the general population in northern Sweden who were occupationally active at baseline and at the 5-year follow-up**

**Variable**	**Baseline (2004)**	**5-year follow-up (2009)**	**P-value** ^**a**^
Age, mean (SD)	43.6 (9.5)	48.6 (9.5)	
SMBQ score, mean (SD)	2.86 (0.91)	2.71 (1.05)	<0.001
SMBQ score > 4.0, N (%)	74/623 (11.8)	82/623 (13.2)	0.37
Marital status, N (%)			
Married or cohabiting	493/609 (81.0)	501/609 (82.3)	
Unmarried, divorced or widowed	116/609 (19.0)	108/609 (17.7)	0.36
Living with children, N (%)			
Yes	362/610 (59.3)	312/610 (51.1)	
No	248/610 (40.7)	298/610 (48.9)	<0.001
Social support (integration), N (%)			
High	389/577 (67.4)	374/577 (64.8)	
Low	188/577 (32.6)	203/577 (35.2)	0.24
Education, N (%)			
University or similar	232/620 (37.4)	236/620 (38.1)	
Other	388/620 (62.6)	384/620 (61.9)	0.45
Socioeconomic index (SEI), N (%)			
Blue-collar	243/621 (39.1)	233/621 (37.5)	
White-collar	320/621 (51.5)	344/621 (55.4)	
Self-employed	58/621 (9.3)	44/621 (7.1)	0.001
Number of work hours, N (%)			
Full-time	510/601 (84.9)	507/601 (84.4)	
Part-time (<35 hours per week)	91/601 (15.1)	94/601 (15.6)	0.76
Demand at work, N (%)			
Low	428/605 (70.7)	405/605 (66.9)	
High	177/605 (29.3)	200/605 (33.0)	0.07
Control at work, N (%)			
High	475/607 (78.3)	470/607 (77.4)	
Low	132/607 (21.7)	137/607 (22.6)	0.65
Job strain, N (%)			
No	563/595 (94.6)	554/595 (93.1)	
Yes	32/595 (5.4)	41/595 (6.9)	0.23
Risk for unemployment, N (%)			
Low	544/603 (90.2)	541/603 (89.7)	
High	59/603 (9.8)	62/603 (10.3)	0.73
New job possibilities, N (%)			
Yes	351/609 (57.6)	293/609 (48.1)	
No	258/609 (42.4)	316/609 (51.9)	<0.001
Perceived economic situation, N (%)			
Satisfied	454/617 (73.6)	405/617 (65.6)	
Dissatisfied	163/617 (26.4)	212/617 (34.4)	<0.001

SMBQ = Shirom Melamed Burnout Questionnaire, score range 1–7.

^a^P-value for changes between 2004 and 2009.

The data from 2009 showed a tendency (non-significant) towards a lower SMBQ mean score in the oldest age group, with a mean score of 2.52, compared with the youngest group with a mean score of 2.79. In the whole study population, the mean SMBQ score was significantly lower for men than for women, (mean values of 2.58 and 2.82 respectively, p < 0.01). However, no interactions were found between the sexes and any of the risk factors that were further investigated with regard to SMBQ score change.

SMBQ score change (the value at year 2009 minus the value at year 2004) for specific age and sex groups are given in Table 2. A significant reduction in burnout level was observed in the age group 50–59 years, and a near significant reduction was seen in the age group 30–39 years. No differences were found between men and women in any of the age groups. The largest decrease in SMBQ score was found in women aged 50–59 years old and in men 30–39 years old.

**Table 2:** **SMBQ score change in different age groups**

**Age (2009)**	**SMBQ score change**	**P-value** ^**a**^	**Sex**	**N**	**SMBQ score change**	**Min – Max**	**P-value** ^**b**^
30–39	−0.16	0.05	Men	61	−0.25	−2.09 – 1.64	
			Women	75	−0.09	−3.64 – 2.95	0.34
40–49	−0.11	0.11	Men	81	−0.16	−2.41 – 2.05	
			Women	88	−0.07	−2.32 – 2.59	0.54
50–59	−0.22	0.002	Men	103	−0.14	−3.22 – 4.04	
			Women	113	−0.28	−3.28 – 3.91	0.33
60–69	−0.09	0.30	Men	55	−0.07	−1.68 – 4.90	
			Women	47	−0.11	−1.77 – 2.27	0.80
All ages	−0.15	<0.001	Men	300	−0.15	−3.22 – 4.90	
			Women	323	−0.15	−3.64 – 3.91	0.99

A negative SMBQ score change indicates reduced burnout level.

^a^P-value regarding SMBQ score change between 2004 and 2009.

^b^P-value regarding SMBQ score change between the sexes in different age groups.

Unemployed persons at follow-up had an SMBQ score change of −0.47, while persons on sick leave reported an increase in SMBQ score (12 persons with temporary sickness benefits had an SMBQ score change of 0.92, and 10 persons with time limited disability pension had an SMBQ score change of 0.24).

### Risk factors and burnout change

Constant exposure to job strain increased the burnout level (Table 3). All persons that experienced constant job strain were actively working persons at follow-up. When job strain was separated into the two dimensions of demands and control, only demands at work were significantly related to an increase in burnout level over the five-year period. Within the demands dimension, transition from low demands in 2004 to high demands in 2009 and constant exposure to high demands were both significantly associated with increased burnout level. The control dimension showed no significant relations. An increased risk of unemployment (low risk in 2004 but a high risk in 2009) was strongly related to an increase in SMBQ score. Although the unemployed persons were excluded from the question of risk of unemployment, 10 unemployed persons stated that their chances of getting a new job had diminished. Links were also found between a worsened self-perceived economic situation (satisfied with ones economic situation in 2004 but dissatisfied in 2009) and an increased burnout level. When combining the four risk factors in one multiple regression analysis (with adjustments for background characteristics), constant job strain, increased risk of unemployment, and worsened self-perceived economic situation remained robust and significantly related to an increased burnout level.

**Table 3:** **Change in SMBQ score in relation to risk factor changes from 2004 to 2009**

**Risk factors**	**N**	**SMBQ change**	**P-value** ^**a**^
Job strain			
Constantly not exposed^ref^	522	−0.18	
Job strain → not exposed	24	−0.02	0.55
Not exposed → job strain	32	−0.05	0.54
Constant job strain	8	0.60	0.01
Demands dimension			
Constant middle-low demands^ref^	330	−0.24	
High demands → middle-low demands	68	−0.42	0.13
Middle-low demands → high demands	91	0.02	0.02
Constant high demands	107	0.14	<0.01
Control dimension			
Constant middle-high control^ref^	410	−0.16	
Low control → middle-high control	57	−0.21	0.54
Middle-high control → low control	61	−0.03	0.36
Constant low control	70	−0.15	0.49
Risk of unemployment			
Constant no risk^ref^	505	−0.19	
Risk → no risk	36	−0.37	0.26
No risk → risk	38	0.37	<0.001
Constant risk	22	0.11	0.17
New job possibilities			
Constant higher chances^ref^	241	−0.20	
Lower chances → higher chances	51	−0.44	0.10
Higher chances → lower chances	108	0.02	0.08
Constant lower chances	198	−0.10	0.36
Self-perceived economic situation			
Constantly satisfied^ref^	335	−0.24	
Dissatisfied → satisfied	71	−0.29	0.86
Satisfied → dissatisfied	121	0.14	<0.001
Constantly dissatisfied	92	−0.07	0.10

A negative SMBQ score change indicates reduced burnout level.

^a^Adjustments for age, sex, socioeconomic index, and social integration.

^ref^This category is considered the reference (unexposed) category in the analysis.

The overall mean SMBQ score change in the cohort was −0.15.

Unemployed persons were not included in analyses regarding job strain, demands, control or job insecurity (risk of unemployment, new job possibilities).

When studying how many risk factors the respondents were exposed to (having a constant unfavourable situation or changing from a favourable situation in 2004 to an unfavourable situation in 2009) with regard to SMBQ score change, it was evident that an increase in the number of risk factors resulted in an increase in SMBQ score (Table 4). The group with no risk factor exposure had the highest decrease in SMBQ score, and those exposed to two or more risk factors had the highest increase.

**Table 4:** **SMBQ score change in the study cohort depending on the number of concurrent risk factors** ^**a**^

**Exposure** ^**a**^	**N**	**SMBQ score change**	**p-value**
0 risk factors ^ref^	203	−0.34	
1 risk factor	232	−0.20	0.11
2 risk factors	144	0.12	<0.001
3-4 risk factors	30	0.32	<0.001

^a^Possible risk factors: job strain, risk of unemployment, new job possibilities, and self-perceived economic situation (having a constant unfavourable situation or going from a favourable situation to an unfavourable position).

^ref^This category is considered the reference (unexposed) category in the analysis.

## Discussion

This five-year prospective cohort study in northern Sweden showed a significant overall decrease in burnout level between 2004 and 2009 in an occupationally active subset of the general population. However, the results indicate a link between constant job strain, an increased risk of unemployment, and a worsened self-perceived economic situation and an increase in burnout level. Furthermore, we found that exposure to a larger number of these risk factors was associated with a larger increase in burnout level.

The decrease in burnout level in the whole cohort was small yet strongly significant. A probable explanation for this is that the cohort had become older. At baseline, the level of burnout was lowest in the oldest age group [28]. However, in the burnout literature there has been some debate about the effects of age on burnout, and conflicting results have been shown [35-37]. The use of different measures of burnout in the studies might be one reason for the varying results. The healthy worker effect [38], which means that the healthier people tend to remain in the actively working population, might also be a plausible explanation for a decreased burnout level with increased age. However, in our study we retained the cohort of people with temporary sickness benefits and people who had become unemployed or had become students when studying the effects of age. We excluded only retired people and persons on disability pension without continued employment.

A previous meta-analysis showed a small negative correlation between years of experience in a field and burnout [37]. This was partially explained by the development of coping strategies among employees who remained in the same occupation or at the same workplace. Employees who do not acquire the necessary coping strategies for a particular job might be more likely to change workplace or occupation or possibly fall ill. Other possible explanations for a reduction in burnout with increasing age might be found in career demands. At a higher age, career goals or general job satisfaction might have been reached. Supervisors might have different expectations for older and younger working persons with regard to certain work tasks and the time required to execute them. Also, changes in private life, such as grown-up children and more free time, might reduce the risk of burnout and exhaustion [39].

The job demand-control model developed by Karasek in the late 1970s explains how work stress emerges and has been the predominant instrument used in studies on the health effects of work stress ever since [33]. According to the theory, high demands can be tolerated if control (decision latitude) is adequately high. The most stressful work situation is job strain, where the demands are high and control is simultaneously low. The complementary job-demand-resources model emphasizes that job demands might turn into stressors when the effort requested is too high leading to insufficient recovery and depleted resources [40]. We found that constant job strain over time was a significant risk factor for increasing burnout, which is consistent with the idea that a poor psychosocial work environment over a long period of time can prey on a person’s resources. This finding is in accordance with previous prospective studies examining the effect of job strain on exhaustion [18,41]. When examining the job strain factor in our study population, we found that demands at work explained most of the changes in burnout, whereas control had no influence. These results differ from previously published data from working populations where low levels of control have been found to be associated with an increased occurrence of burnout or emotional exhaustion [20,30]. In the present follow-up, the proportion of female subjects exposed to low control at baseline was larger among non-responders than among participants. This might have reduced the chance of observing any associations with the control dimension. However, regarding demands at work specifically, an overall robust relation to future burnout has been reported in the majority of earlier studies [13,30,42], and this is also supported by our findings. In the majority of earlier studies baseline burnout levels have not been taken into account. In the present study, information on both risk factors and outcome at baseline as well as follow-up is available and was used in the analyses, and this might have increased the reliability of our results. Our findings are also largely consistent with the results in a Dutch prospective study on a working population where risk factors and exhaustion were established both at baseline and follow-up [30].

During the study period (year 2004 to 2009) there was a decline in the world economy which also affected the Swedish population. One of the factors measuring job insecurity, namely, the risk of unemployment, showed an effect on burnout level in a short time perspective with a change to a worsened self-perceived situation being related to an increase in burnout.

Similar results have been found for men experiencing downsizing at work during the last two years [20]. It has been suggested that the labour culture has shifted from a loyal relationship between employers and employees to a more insecure situation regardless of length of employment [25]. Feelings of insecurity, and not just actual redundancies with dismissals, can occupy a person’s mind and make them lose focus. Because of the economic market crisis that occurred during the period between baseline and follow-up, it is possible that those who expressed feelings of job insecurity in 2009 felt more hopelessness in terms of gaining new employment with the overall rise in unemployment figures. New job possibilities did not significantly relate to a change in burnout level, but the respondents in the category “increased new job possibilities” had the greatest decrease in SMBQ score. This suggests that a newly acquired belief in one’s employability might buffer feelings of burnout.

Because adjustments have been made in the present study regarding socioeconomic status, the significant association observed between an increase in burnout and a worsened self-perceived economic situation is not necessarily equivalent with having a lower income. The variable “perceived economic situation” was created originally in the MONICA study to measure satisfaction with the personal financial situation, and not the actual economic status or income. The finding that those who experienced a deteriorated economic situation during the study time also reported increasing burnout levels indicates that subjective economic difficulties even in a short time perspective might have an impact on burnout. One explanation for this could be that people who started to worry about their economic situation during the five years in between the measuring points had loans or other financial commitments that were affected by the economic market decline at the end of the last decade.

In the multiple regression analysis, exposure to the three risk factors of constant job strain, increased risk of unemployment, and a worsened (dissatisfied) self-perceived economic situation all remained significant, which indicates that these risk factors have additive effects. In addition to indicating a stable relation between risk factors and outcome, this also suggests that each of these three significant risk factors independently affect the burnout level regardless of the presence of the others. For example, the effect that self-perceived economic situation has on burnout level is not enhanced or reduced by increased job insecurity.

Our findings show that an accumulation of risk factors corresponded to higher SMBQ scores, and burnout level increased with each additional risk factor the person was exposed to. Although the individual risk factors did not seem to influence each other much in the multiple linear regression analysis, when put together they might cause a downward spiral. Being exposed to only one risk factor is manageable, but if exposure to additional risk factors occurs it might wear a person down and increase burnout level.

### Study strengths and limitations

As described above, the same questionnaires were filled out at baseline and at follow-up. A point to consider is that the answers to the questions are self-perceived and mirror the state of the respondents at two specific points in time. Nevertheless, the fact that changes are studied can help reduce the problem of negative affectivity. Still, common method variance might be a problem because all data variables were collected by the same questionnaire at both points in time, and this would most probably inflate the observed associations.

The SMBQ has been shown to correlate well with other burnout instruments, such as the Emotional Exhaustion subscale of the Maslach Burnout Inventory (MBI) and the Pines Burnout Measure [43]. In Sweden, the SMBQ has also been studied in relation to clinical criteria for the diagnosis Exhaustion Disorder, which is closely linked to burnout, and strong associations have been found with the self-rated Exhaustion Disorder (s-ED) instrument [10]. The SMBQ is based on a generic burnout concept which implies that factors outside working life also might have been of importance for the burnout level. In this study we did not control for personal life events. The relations between work-specific factors and the SMBQ score found in this study might have been attenuated due to this fact. However, working and private lives are becoming increasingly integrated and might influence each other in many ways.

By using a continuous SMBQ variable, we have been able to study smaller changes in the population. It has been possible to register changes along the whole spectrum of the SMBQ scale and not only those that cross a cut-off point. One difficulty with handling the data in this manner is the respondents who were at the far ends of the scale at the first measuring point. These respondents could mainly move in only one direction, namely towards the middle of the scale. We cannot draw conclusions regarding people’s development of clinical burnout syndromes; we only present fluctuations of SMBQ scores in the population.

The risk factors evaluated in this longitudinal study consisted of demands, control, and strain at work; job insecurity; and self-perceived economic situation, all of which have been found to be important for well-being and health in previous studies [19-23,28]. Job insecurity and self-perceived economy were assessed in our study by single-item questions, which have been used in repeated MONICA surveys in northern Sweden and were considered to have good face validity. A strength of this study is the measurement of these risk situations with the same questions at baseline and follow-up that enables analyses that take changes of these situations into consideration. However, a follow-up time of five years is long and might imply many ups and downs in people’s lives.

The fact that there were differences at baseline between the female responders and female non-responders could have resulted in an under- or overestimation of risk. However, the response rate of 70% can be considered high. In contrast to many other burnout studies, we present an occupationally active subset of the general population that is made up of people with many different types of occupations in different types of work environments.

## Conclusions

In this study a decrease of burnout over a five-year period was detected in an occupationally active subset of the general population. The results of this study add to previous findings by showing that changes in work-related risk factors and self-perceived economic situation affect changes in burnout level. Furthermore, an accumulation of risk factor exposure seems to increase burnout level. This suggests that a little strain can be manageable, but people’s stamina diminishes with each additional risk factor they are exposed to. These risk factors can be amended and indeed prevented. Improved working conditions might reduce psychological ill health and, consequently, sickness absences.
